# Endoscopic esophageal foreign body removal among children at Lubaga Hospital, Kampala, Uganda

**DOI:** 10.4314/ahs.v23i3.27

**Published:** 2023-09

**Authors:** Michael Okello, Sabrina Bakeera-Kitaka, Ponsiano Ocama, Esther Patience Nabwire, Dave Darshit, Christine Namata, Annah Ainembabazi Tinka

**Affiliations:** 1 Department of Anatomy, Makerere University College of Health Sciences; 2 Department of Medicine, Makerere University College of Health Sciences; 3 Lubaga Hospital, Kampala, Uganda

**Keywords:** Endoscopy, esophagus, foreign body

## Abstract

**Background:**

Diagnostic upper gastrointestinal endoscopy involves examination of the lining of the esophagus, stomach and part of the duodenum. Interventional endoscopy in addition to evaluating the upper gastrointestinal tract to make a diagnosis, also offers a treatment benefit. Traditionally, esophageal foreign bodies (FBs) in Uganda were removed using rigid endoscopy. We therefore report an emerging trend of using flexible endoscopy to remove these FBs.

**Objective:**

To describe participant characteristics and endoscopic findings among children who underwent esophageal FB removal in Lubaga Hospital in Kampala.

**Methods:**

This was a retrospective review of endoscopy reports for children who underwent endoscopic esophageal FB removal at Lubaga Hospital from December 2014 to March 2022.

**Results:**

Overall, 61 symptomatic children underwent this procedure. The majority of the FBs were removed by flexible endoscopy (n=55, 90.16%). The mean age of the participants was 7.88 (SD=2.12) years old. The majority of the children were females (72.13%) and coins were the most ingested FBs (84%), others included steel crucifix, nails etc. The upper esophageal sphincter was the commonest site for FB impaction (74%).

**Conclusion:**

We report high success rates of 90.16% for endoscopic removal of impacted esophageal foreign bodies among Ugandan children using the now widely available flexible endoscopy.

## Introduction

Foreign body (FB) ingestion is a commonly encountered clinical problem at the emergency room especially in the pediatric population. Ingestion of these foreign bodies is commonly accidental, intentional ingestion is frequent amongst the adolescent group 1. Foreign body (FB) impaction accounts for 4% of emergency endoscopies in clinical practice 2.

A study by Nakku et al. suggests that aero digestive FBs are the most recurrent emergencies seen at the ENT department at Mbarara regional referral hospital. While the majority of FBs pass through the GIT without causing harm, some necessitate intervention to reduce morbidity and mortality. One of the main challenges is FB impaction. Symptoms of foreign body impaction include dysphagia, odynophagia, sensation of objects stuck in the throat, drooling, vomiting 1, 3, 4.

Intervention for ingested foreign bodies should be timely as there is potential to cause respiratory complications, intestinal obstruction, esophageal erosions and perforation 5. Upper GI endoscopy is both a diagnostic and interventional module in management of FBs. Flexible endoscopy (FE) is recommended as the first-line therapeutic option because it can be performed under sedation, is cost-effective, and is well tolerated 2.

Endoscopic management of foreign bodies offers advantages such as low cost, minimal invasiveness, reduced morbidity mortality and high removal success rates 6.

A number of these procedures have been performed at our hospital. We aimed to describe participant characteristics and endoscopic findings among children who underwent esophageal foreign body removal in Lubaga Hospital in Kampala, Uganda.

We hope that the results will inform clinicians and hospitals on the necessity to provide this procedure for patients with suspected foreign body ingestion.

## Methods

### Study Design

This was a retrospective review of endoscopy reports for children of ages 1 to 12 years who underwent endoscopic esophageal foreign body removal at Lubaga Hospital from December 2014 to March 2022.

### Study site and setting

The study was conducted at Lubaga Hospital, a private not for profit hospital located in Lubaga division of Kampala capital city of Uganda. This is the second oldest hospital in Uganda founded by the catholic missionaries in 1899. The hospital has an endoscopy unit, under the Surgery department that runs both inpatient and outpatient services, as well as diagnostic and interventional services. The hospital acquired an endoscopy unit in the year 2014 and also acquired a C-arm. The hospital performs both diagnostic and some therapeutic procedures including esophageal stenting, variceal banding, ERCP, foreign body removal and a number of other procedures both for children and adults. In addition to upper GIT scope, lower GI diagnostic and therapeutic procedures including colonoscopies and polypectomies are performed. The unit receives referrals from within the hospital and other hospitals within Kampala and sometimes beyond Kampala City, and an average of 60 endoscopy procedures are performed in a month.

This study was approved by Makerere University College of Health Sciences, School of Medicine Research and Ethics Committee at it's 116 convened meeting held on 11/03/2021. Ref: Mak-SOMREC-2021-71. Informed written consent was obtained from the parents/guardian for publication and accompanying images. A copy of the written consent is available for review by the Editor-in-Chief of this journal on request.

### Data Collection and analysis

A data abstraction tool collected information on demographics (age, gender, and presenting complaints), endoscopic (foreign body type, location of foreign body), interventions.

Data was summarized using descriptive measures. Demographic characteristics were summarized by standard descriptive summaries (means and standard deviations for age and percentages for categorical variables such as gender).

## Results

### Participants' characteristics

All records reviewed belonged to children that underwent endoscopy for confirmed /suspected FB ingestion. Overall, 61 children underwent the procedure under local anesthesia and sedation. The mean age of the children was 7.88 ± 2.12 years. Majority were below the age of five years old 43 (70.49%) were mostly of female gender 44 (72.13%). Table 1. All the 61 children referred for endoscopic foreign body removal were symptomatic; with one or more of features like dysphagia, drooling saliva, chest pain etc. Table 2.

**Table 1:** Demographic characteristics of patients undergoing endoscopic foreign body removal

Demographic Characteristic		Number of patients (%)
Age (years)	<5	43 (70.49)
	6-10	13 (21.31)
	>10	5 (8.20)
Gender	Females	44 (72.13)
	Males	17 (27.87)

**Table 2:** Types of the foreign bodies and their location

Variable	Number of patients (%)
**Clinical Presentation**	
Symptomatic*	61 (100)
Asymptomatic	00 (00)
**Type of foreign body**	
Coin	51 (83.61)
Metal + nails	2 (3.28)
Others	8 (13.12)
**Distance from incisor**	
Middle 1/3 esophagus	15 (24.59)
Upper esophageal sphincter	46 (74.41)

* Symptoms included one or more of dysphagia, drooling saliva, chest pain etc

### Endoscopic findings of the foreign body patients

Coins were the most frequently ingested foreign body (n=51, 83.61%), sharp objects consisted of metal pieces and nails which were ingested by suicidal patients and accounted for 3.28 % (n=2). Other blunt objects included batteries, plastic bottle tops and food boluses Overall, 46 children (74.41%) had upper esophageal FB impaction at the level of the upper esophageal sphincter. There were 15 impactions in the middle esophagus (24.59%). Table 2.

### Procedure outcome for foreign bodies

55 of 61 children had the foreign bodies removed by flexible endoscopy (90.16%) while 5 had the foreign bodies pushed to the stomach for expectant excretion per annum In one child, no intervention was done as no foreign body was identified in the esophageal lumen during the procedure. Table 3.

**Table 3:** endoscopic interventions done

Intervention	Number of patients (%)
Removal by flexible endoscopy	55 (90.16)
Pushed to stomach	5 (8.20)
No Intervention	1 (1.64)

## Discussion

FB injuries in the upper digestive tract continue to be a common health problem in children. In our study, the majority of cases involved children below the age of 5, showing similarities with the results of the ESFBIs study and with Lin's study 7, 8. This similar finding was reported by Kalra, who found common occurrences of foreign bodies in those between 0-5 years 1. This could be accidental or could be attributed to the curiosity of the children 10. We also found that foreign bodies occur more in females (79.17%) than in males. However, Kalra et al. reported foreign bodies in more males than females.

With regard to foreign body types, fish bones, metal objects such as batteries and coins, and broken tooth fragments are the most frequently ingested foreign bodies according to scientific literature 9, 11, 12. In our study, coins were by far the most frequent foreign body ingested. This was in agreement with a number of previous studies that reported similar findings 1, 3, 4. Coin ingestion dominates the literature on esophageal foreign bodies owing to accessibility and availability to the general and pediatric population as a legal tender, and adults often unwisely give young children coins for various reasons.

**Figure 1:**
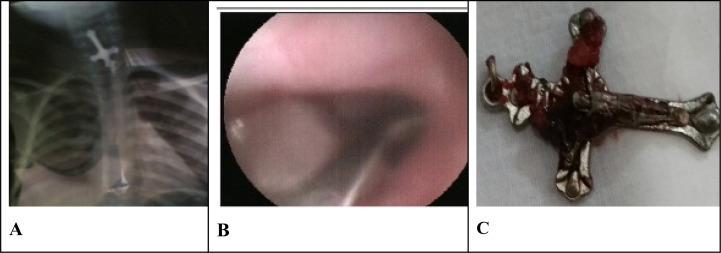
(A) chest X-ray, (B) endoscopic image (C)same crucifix after endoscopic removal from a 6-year-old girl

The majority of swallowed foreign bodies pass harmlessly and spontaneously through the gastrointestinal tract (GIT) 7, but in case of lodgment or toxicity of the object, the FBs must be rapidly identified and removed. Prompt endoscopic intervention is the gold standard for all complicated or high-risk situations with relevance to sharp and pointed FBs. Some foreign bodies can be sharp and may perforate the esophagus if not carefully removed. We removed a crucifix that was swallowed by a 6-year-old girl safely without complications figure1. The most frequent lodgment site described in literature is the upper esophageal sphincter found around the region of the cricopharyngeus muscle 9, 13, 14, a similar finding in our study that revealed most objects lodged at the cricopharyngeal region.

Management of esophageal FBs is determined by the type of object, its lodgment site, and the symptoms at the time of presentation. A study by Waltzman et al. in 2005 concluded that assuming the child is asymptomatic, the ingestion is recent, and the child has no underlying esophageal or tracheal abnormality, an 8- to 16-hour period of observation is appropriate management in children with esophageal coins 15. In another study however, he recommended immediate removal via endoscopy in symptomatic patients with an esophageal coin 16. Rigid esophagoscopy remains the most popular technique for removal of the esophageal foreign bodies. Others include flexible fiberoptic esophagoscopy, Foley catheter technique, and esophageal bougienage. The technique of pushing the FB into the stomach with a bougie has also been advocated.

In this study 55 of the 61 children reviewed had removal by flexible endoscopy done as they presented with one or more symptoms ranging from dysphagia, drooling saliva and retrosternal chest pain. The esophageal FB was pushed to the stomach in 5 of children. And one child despite having symptoms was managed conservatively after endoscopic findings revealed no esophageal FB but just scratch marks most likely from a fish bone that had already been spontaneously expelled. This kind of intervention (flexible endoscopic FB removal) is done to relieve obstruction in the esophagus and upper airway. This intervention was similarly reported by Chinski et al. in 2010 17. Removal is vital because delay increases the risk of complications, including perforation, and decreases the likelihood of successful removal. In Uganda currently, most tertiary level hospitals at least have an endoscopy tower mostly used for diagnostic endoscopy. Future and current plans to advocate for more training in interventional endoscopy so that such common cases can be safely managed at all levels should be supported.

## Conclusion

We report high success rates of 90.16% for endoscopic removal of impacted esophageal foreign bodies among Ugandan children using the now widely available flexible endoscopy.

Our findings will inform clinicians and hospitals on the necessity to provide this procedure for patients with suspected foreign body ingestion in Uganda.
